# Nicotine dependence, major smoking-related disease, and sustained smoking cessation among middle-aged and older adults in China: A secondary analysis of the China Health and Retirement Longitudinal Study, 2011–2018

**DOI:** 10.18332/tid/240543

**Published:** 2026-10-22

**Authors:** Liyong Shi, Miaoxiang Chen, Youjuan Wang, Tanwei Liu, Lianshun Lin, Fanbin Zhong, Qidong Chen, Xiaoyang Chen

**Affiliations:** 1 Department of Pulmonary and Critical Care Medicine, the Second Affiliated Hospital of Fujian Medical University, Quanzhou, China; 2 Department of Nursing, the Second Affiliated Hospital of Fujian Medical University, Quanzhou, China

**Keywords:** smoking cessation, nicotine dependence, heaviness of smoking index, chronic disease, China

## Abstract

**INTRODUCTION:**

Nicotine dependence may impede cessation, whereas major smoking-related disease may motivate quitting. We investigated their associations with sustained smoking cessation among middle-aged and older adults in China.

**METHODS:**

We analyzed four waves (2011, 2013, 2015, and 2018) of the China Health and Retirement Longitudinal Study (CHARLS). The sample included 2148 baseline cigarette smokers aged ≥45 years with complete Heaviness of Smoking Index (HSI) data and concordant smoking status in 2015 and 2018. Sustained cessation required no current smoking at both waves. The HSI scores (0–6) combined daily cigarette consumption and time to the first cigarette after waking. Major disease comprised heart disease, stroke, chronic lung disease, or cancer –multivariable logistic regression estimated mutually adjusted associations. Sensitivity analyses addressed missing data, attrition, outcome definition, nonlinearity, and smoking transitions.

**RESULTS:**

Overall, 337 participants (15.7%) achieved sustained cessation. Each 1-point higher HSI was associated with lower odds of sustained cessation (AOR=0.689; 95% CI: 0.631–0.752), whereas major disease was associated with higher odds (AOR=1.494; 95% CI: 1.087–2.054). There was no HSI × disease interaction (p=0.897). In a four-knot restricted cubic spline model, HSI was associated with sustained cessation overall (Wald χ^2^=121.08; df=3; p<0.001) and nonlinearly (Wald χ^2^=28.40; df=2; p<0.001). Relative to HSI=2, AOR was 3.47 (95% CI: 2.51–4.80) at HSI=0, and 0.97 (95% CI: 0.56–1.70) at HSI=6. Sensitivity analyses yielded similar estimates. Under a stricter three-wave definition, the corresponding AORs for HSI and major disease were 0.599 (95% CI: 0.486–0.739) and 2.284 (95% CI: 1.052–4.957), respectively.

**CONCLUSIONS:**

Among participants with concordant smoking status in 2015 and 2018, greater nicotine dependence was associated with lower odds of sustained cessation versus persistent smoking. In contrast, major smoking-related disease was associated with higher odds. Chronic disease care may provide an opportunity for cessation support, but the findings do not establish causality. Brief HSI assessment may identify smokers needing intensive support.

## INTRODUCTION

Smoking cessation in midlife and later life remains an important opportunity to reduce tobacco-related disease burden^1^. This is particularly relevant in China, where smoking remains concentrated among middle-aged and older men and socioeconomically disadvantaged groups^2,3^. Prospective evidence in Chinese adults has linked smoking to cardiovascular, respiratory, malignant, and other diseases^4^. However, realizing the population health benefits of cessation depends in part on maintaining abstinence. In the China Health and Retirement Longitudinal Study (CHARLS), 13.3% of participants who reported quitting in 2013 were smoking again by 2015^5^. A former-smoker classification based on a single interview may therefore reflect a transient change rather than sustained cessation.

Two factors may shape whether smoking cessation is sustained. The first factor is nicotine dependence, operationalized using the Heaviness of Smoking Index (HSI). The HSI combines cigarettes smoked per day with the time to the first cigarette after waking, capturing tobacco consumption and the urgency of smoking after overnight abstinence^6^. Higher HSI scores are associated with a lower probability of smoking cessation^7^, which includes smokers with serious illness^8^. The second factor is major smoking-related disease. A diagnosis of such a disease may make the health consequences of smoking more immediate, increase contact with healthcare services, and create opportunities for cessation advice. In CHARLS, incident diagnoses of cancer, heart disease, or stroke were followed by reductions in smoking, although these associations attenuated with increasing time since diagnosis^9^. Nevertheless, persistent smoking remains common among Chinese adults with smoking-related morbidity^10^. Illness may therefore strengthen the motivation to quit without overcoming the nicotine dependence that makes sustained abstinence difficult.

These evidence streams have generally developed separately. Previous CHARLS research found that fewer cigarettes per day and a longer interval before the first morning cigarette predicted quitting, but the two HSI components were examined separately and follow-up ended in 2015^5^. Other studies have focused on incident disease diagnoses^9^, social participation among smokers with morbidity^10^, or socioeconomic patterning of cessation^11^. More recent work classified nicotine dependence using cigarette consumption alone and examined chronic disease as the outcome rather than sustained cessation^12^. Consequently, it remains unclear how a validated two-item dependence measure and major smoking-related disease jointly relate to repeated non-smoking status across later CHARLS waves. Modeling both factors together allows estimation of their mutually adjusted associations, while repeated smoking assessments help distinguish sustained cessation from a transient change in smoking status.

The primary aim of this study was to investigate whether baseline nicotine dependence, measured using HSI, and major smoking-related disease were associated with sustained smoking cessation among middle-aged and older adults in China using four waves of CHARLS. The primary comparison contrasted participants reporting no current smoking at both the 2015 and 2018 interviews with those reporting current smoking at both interviews. We hypothesized that, when included in the same model with prespecified covariates, higher HSI would be associated with lower odds of sustained cessation and higher odds of major disease. We also assessed nonlinearity, a stricter three-wave definition of sustained cessation, missing data and attrition, alternative smoking transitions, the HSI × disease interaction, and exploratory variation by sex and birth cohort.

## METHODS

### Study design and data source

This study was a secondary analysis of data from CHARLS, an ongoing, nationally representative longitudinal survey of adults aged ≥45 years in China and their spouses. The original cohort was selected using multistage probability sampling from 150 county-level units and 450 village-level communities. Its design and field procedures have been described previously^13^. We used data from the 2011 baseline and follow-up interviews conducted in 2013, 2015, and 2018. The present analysis focused on longitudinal associations among eligible participants rather than nationally representative estimates of smoking cessation. The Biomedical Ethics Review Committee of Peking University approved the CHARLS protocol (IRB00001052-11015), and all participants provided written informed consent. The present study used de-identified secondary data in accordance with the CHARLS data-use agreement.

### Participants

Participants were eligible for the primary analysis if they were aged ≥45 years, reported current cigarette smoking at baseline, had complete data for both HSI components, and had concordant smoking status in 2015 and 2018. We identified baseline current cigarette smoking using the harmonized CHARLS indicator R1SMOKEN=1 among participants with a completed baseline interview (R1IWSTAT=1). At each follow-up, RwSMOKEN=1 indicated current smoking and RwSMOKEN=0 indicated current non-smoking; missing values were retained as missing.

At baseline, 4762 participants aged ≥45 years reported current smoking. Of these, 4137 reported smoking hand-rolled, filtered, or unfiltered cigarettes. Among them, 3513 had valid data on both cigarettes smoked per day and time to the first cigarette after waking, allowing calculation of the HSI. The primary analysis further required smoking-status data in both 2015 and 2018 and included participants who reported either current smoking at both interviews or no current smoking at both interviews. The resulting analytic sample comprised 2148 participants, including 1811 with persistent smoking and 337 with sustained cessation. Participants with discordant smoking status in 2015 and 2018 were excluded from the primary binary comparison but were not considered to have missing outcomes. We classified them as relapse or late cessation and retained them for secondary analyses (Figure 1).

**Figure 1:**
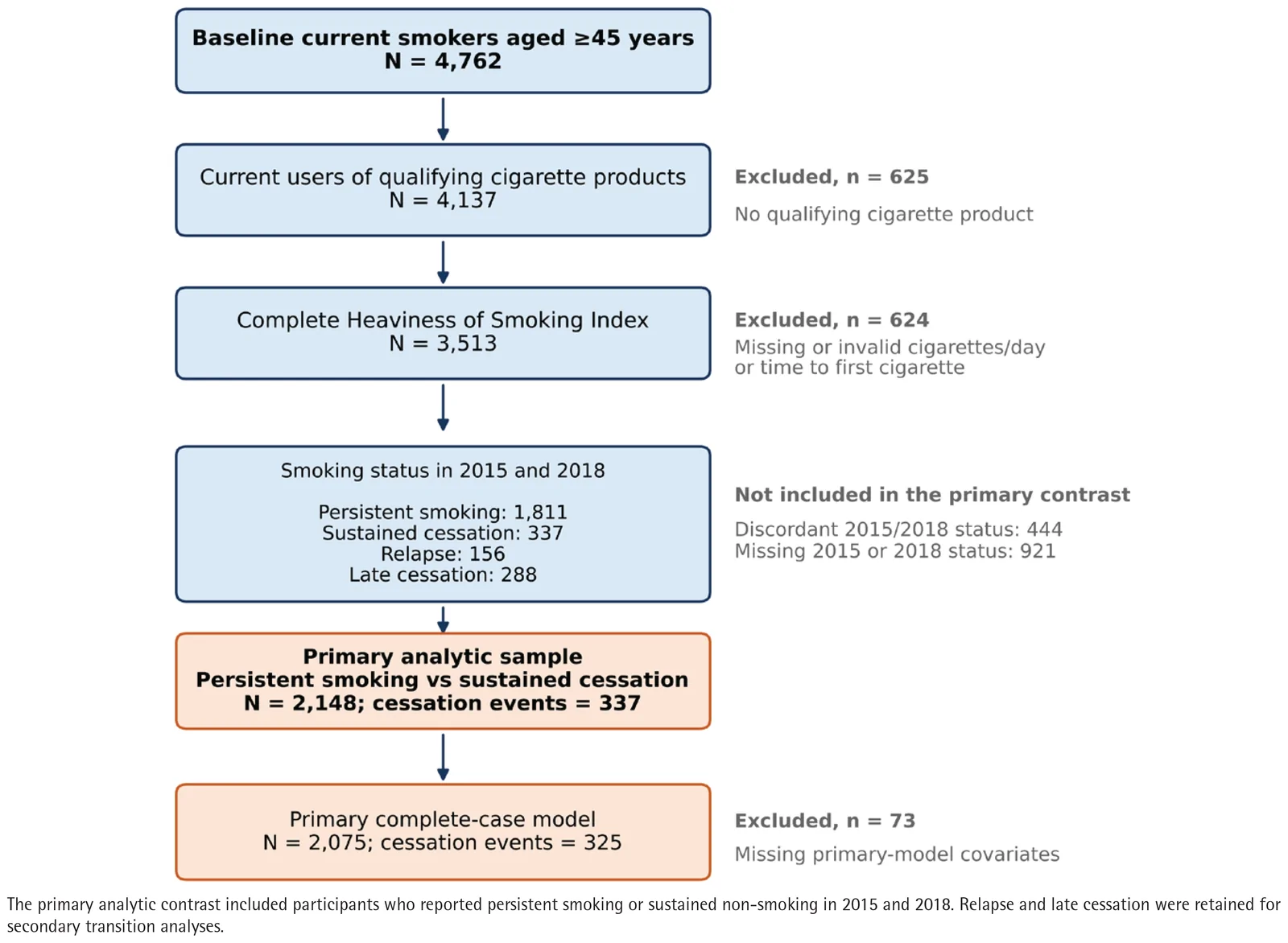
Participant selection and smoking-transition groups in CHARLS, 2011–2018 (baseline smokers with complete HSI data, N=3513; primary analytic sample, N=2148)

### Heaviness of Smoking Index

The HSI was scored according to the original algorithm^6^. Daily cigarette consumption contributed 0 points for 1–10 cigarettes, 1 point for 11–20, 2 points for 21–30, and 3 points for at least 31. Time to the first cigarette after waking contributed 3 points for ≤5 minutes, 2 points for 6–30 minutes, 1 point for 31–60 minutes, and 0 points for >60 minutes. The two component scores were summed to yield a total score ranging from 0 to 6. HSI was modeled as a continuous variable in the primary analysis. Potential nonlinearity was assessed using the four-knot logistic restricted cubic spline model described below.

### Major smoking-related disease

Major smoking-related disease was defined *a priori* as at least one self-reported physician diagnosis of heart disease, stroke, chronic lung disease, or cancer at baseline. The composite was defined only when all four component indicators were observed; if any component was missing, the composite was set to missing. A binary indicator was used because cancer and stroke were uncommon in the analytic sample and disease-specific models would have been unstable. To distinguish this indicator from broader multimorbidity, models also included a count of ten other baseline conditions: hypertension, diabetes, dyslipidemia, kidney disease, psychiatric problems, arthritis, asthma, memory-related disease, digestive disease, and liver disease. The term ‘smoking-related’ reflects established population-level associations with tobacco and does not imply that smoking caused an individual participant’s disease.

### Smoking outcomes

All participants reported current smoking at baseline in 2011. Sustained cessation, the primary outcome, was defined as non-smoking in both 2015 and 2018, with persistent smoking at both interviews as the reference. A stricter sensitivity definition required non-smoking in 2013, 2015, and 2018 and used smoking at all three follow-up interviews as the reference. Four mutually exclusive smoking patterns during 2015–2018 were also defined: persistent smoking, sustained cessation, relapse (non-smoking in 2015 followed by smoking in 2018), and late cessation (smoking in 2015 followed by non-smoking in 2018). Relapse and late cessation were each compared with persistent smoking in separate secondary logistic regression models. These models were used to describe transition-specific associations, and coefficients were not formally compared across models.

### Covariates and conceptual framework

Baseline covariates were prespecified based on subject-matter knowledge and a directed acyclic graph developed following established guidance^14^ and presented in Supplementary file Figure S1. The primary adjustment set comprised baseline age, sex, rural or urban residence, education level, partnered status, current alcohol use, and the number of other chronic conditions. Baseline age was obtained from the Harmonized CHARLS wave-1 age variable, which represents age in years at the 2011 interview.

Education level was categorized as lower than primary school, primary school, middle school, or high school and higher. Partnered status was dichotomized as married/partnered versus not partnered. Household income had substantial missing data and was added in a secondary model rather than the primary complete-case model. A further secondary model included depressive symptoms measured using the 10-item Center for Epidemiologic Studies Depression Scale (CESD-10) and body mass index. Self-rated health was summarized descriptively but was not included in the primary adjustment set because it may reflect symptoms and perceived consequences of established disease, making its causal position ambiguous. Because HSI and major disease may share common causes and may also be causally related to one another, coefficients from models containing both variables were interpreted as conditional, mutually adjusted associations rather than total causal effects.

### Statistical analysis

Baseline characteristics were summarized overall and by primary outcome. Continuous variables were reported as means with standard deviations (SDs) or medians with interquartile ranges (IQRs), as appropriate. Categorical variables were reported as frequencies and percentages. Percentages were calculated using non-missing denominators. Between-group p-values in Table 1 were considered descriptive and were not interpreted as tests of balance or used for covariate selection.

**Table 1:** Baseline characteristics by sustained smoking-cessation status in CHARLS, 2011–2018 (N=2148)

*Characteristics*	*Overall* *(N=2148)* *n (%)*	*Persistent* *smoking (N=1811)* *n (%)*	*Sustained cessation* *(N=337)* *n (%)*	*p*
**Sociodemographic**				
Age (years), mean (SD)	56.9 (8.1)	56.5 (8.0)	59.2 (8.4)	<0.001
Female sex	179 (8.3)	150 (8.3)	29 (8.6)	0.929
Rural residence	1373 (63.9)	1153 (63.7)	220 (65.3)	0.613
Partnered	1981 (92.2)	1665 (91.9)	316 (93.8)	0.298
Current alcohol use	1302 (60.6)	1115 (61.6)	187 (55.5)	0.042
Annual household income (RMB), median (IQR)	12804 (2720–33475)	13090 (2960–33325)	10750 (2390–34065)	0.612
**Education level**				
Lower than primary school	672 (31.3)	558 (30.8)	114 (33.8)	0.420
Primary	521 (24.3)	438 (24.2)	83 (24.6)	
Middle school	620 (28.9)	535 (29.5)	85 (25.2)	
High school or higher	335 (15.6)	280 (15.5)	55 (16.3)	
**Heaviness of Smoking Index**				
Low (0–1)	873 (40.6)	643 (35.5)	230 (68.2)	<0.001
Moderate (2–4)	1001 (46.6)	920 (50.8)	81 (24.0)	
High (5–6)	274 (12.8)	248 (13.7)	26 (7.7)	
Mean (SD)	2.4 (1.9)	2.6 (1.9)	1.4 (1.8)	
**Smoking status**				
Cigarettes smoked per day, median (IQR)	20 (10–20)	20 (10–20)	10 (5–20)	<0.001
First cigarette within 30 minutes after waking	1080 (50.3)	993 (54.8)	87 (25.8)	<0.001
**Health status**	**n/N (%)**	**n/N (%)**	**n/N (%)**	
Major smoking-related disease	375/2128 (17.6)	290/1793 (16.2)	85/335 (25.4)	<0.001
Heart disease	186/2138 (8.7)	143/1801 (7.9)	43/337 (12.8)	0.006
Stroke	34/2142 (1.6)	26/1805 (1.4)	8/337 (2.4)	0.307
Chronic lung disease	202/2142 (9.4)	155/1805 (8.6)	47/337 (13.9)	0.003
Cancer	6/2143 (0.3)	4/1808 (0.2)	2/335 (0.6)	0.527
Fair or poor self-rated health	1207/1811 (66.6)	1007/1526 (66.0)	200/285 (70.2)	0.191
Other chronic conditions (count), median (IQR)	1.0 (0.0–2.0)	1.0 (0.0–1.0)	1.0 (0.0–2.0)	0.005
CESD-10 score, median (IQR)	6.0 (3.0–10.0)	6.0 (3.0–10.0)	6.0 (3.0–10.0)	0.866
Body mass index (kg/m^2^), mean (SD)	22.9 (3.6)	22.8 (3.6)	23.3 (3.4)	0.021

Percentages were calculated using variable-specific non-missing denominators. For major smoking-related disease, denominators were 2128 overall, 1793 for persistent smoking, and 335 for sustained cessation; the composite was set to missing if any component indicator was missing. For fair or poor self-rated health, denominators were 1811, 1526, and 285, respectively. Household income was observed for 1259 participants and missing for 889 (41.4%; 760 with persistent smoking and 129 with sustained cessation). Between-group p-values are descriptive and were not used for covariate selection. CESD-10: 10-item Center for Epidemiologic Studies Depression Scale. IQR: interquartile range. RMB: Chinese Renminbi. SD: standard deviation. CHARLS: China Health and Retirement Longitudinal Study.

We fitted multivariable logistic regression models for sustained cessation and reported adjusted odds ratios (AORs) with 95% confidence intervals (CIs). Complete-case CIs and hypothesis tests used sandwich standard errors robust to clustering within CHARLS communities. Model 1 included HSI, major smoking-related disease, the other chronic-condition count, age, and sex. Model 2, the prespecified primary model, additionally included residence, education level, partnered status, and alcohol use. Models 1 and 2 were estimated in the same complete-case sample (N=2075; 325 sustained-cessation events) to permit direct comparison of coefficients across the nested specifications. Models 3 and 4 were secondary extensions of Model 2. Model 3 added inverse-hyperbolic-sine-transformed household income, and Model 4 added CESD-10 score and body mass index.

We modeled the association between HSI and sustained cessation using logistic restricted cubic spline regression with four knots at HSI values 0, 1, 3, and 6, corresponding to the empirical 5th, 35th, 65th, and 95th percentiles. HSI=2, the sample median, was the reference. The spline model used the same complete-case sample and covariates as Model 2. Wald chi-squared tests assessed the overall and nonlinear components, and adjusted odds ratios with 95% confidence intervals were estimated across the observed HSI range. A product term between linear HSI and major disease was added to the primary model to evaluate multiplicative interaction.

Sensitivity analyses addressed missing data, attrition, and outcome definition. Multiple imputation by chained equations used 20 imputed datasets and 20 iterations under a missing-at-random assumption conditional on variables included in the imputation models. Although this assumption was considered plausible given the observed predictors included, dependence on unmeasured factors could not be excluded. Binary disease indicators were imputed using logistic regression. Household income, CESD-10 score, and body mass index were imputed using predictive mean matching. The major disease indicator and other chronic-condition counts were reconstructed in each completed dataset. Regression coefficients and covariance matrices were combined using Rubin’s rules^15,16^. Multiple imputation assumed that data were missing at random, conditional on the variables included in the imputation models. This assumption was considered plausible because missingness in income and health variables could be related to observed demographic, smoking, disease, and socioeconomic characteristics; however, it cannot be verified from the observed data, and bias may remain if missingness depends on unobserved values after conditioning on the included variables. The attrition analysis estimated the probability that the primary outcome was observed using baseline model variables. Stabilized inverse-probability-of-observation weights were then constructed and truncated at the 1st and 99th percentiles.

Effect modification by sex and birth cohort was examined exploratorily. Birth year was categorized as 1945 or earlier, 1946–1955, and 1956–1966. Global Wald tests were used to evaluate product terms. No adjustment for multiple testing was applied to these exploratory analyses. Sex-stratified estimates were interpreted cautiously because women constituted a small proportion of baseline smokers. Analyses were conducted using R version 4.6.1 with the *dplyr*, *readr*, *sandwich*, *lmtest*, *rms*, *ggplot2*, and *mice* packages^17^. Variable-construction and analysis scripts were archived before manuscript revision. Analyses were unweighted because the target estimand was a conditional association within the eligible longitudinal cohort rather than a nationally representative cessation rate. All statistical tests were two-sided. Reporting followed the STROBE recommendations^18^.

## RESULTS

### Participant flow and baseline characteristics

Among 3513 baseline smokers with a complete HSI, 1811 were persistent smokers in 2015 and 2018, 337 met the definition of sustained cessation, 156 had a relapse pattern, and 288 had a late-cessation pattern. The remaining 921 participants could not be classified because smoking-status data were unavailable for one or both follow-up interviews. The primary sample therefore comprised 2148 participants. Seventy-three participants had missing covariates in the primary model, leaving 2075 participants and 325 sustained-cessation events for complete-case inference.

The mean age of the primary sample was 56.9 years (SD=8.1); 63.9% lived in rural areas, and 8.3% were women. The mean HSI was 2.4. Scores were 0–1 in 40.6%, 2–4 in 46.6%, and 5–6 in 12.8% of participants. Major smoking-related disease was present in 375 of 2128 participants (17.6%) with complete disease information. Of these, 73 had missing covariates in the primary model, leaving 2075 participants and 325 sustained-cessation events for Models 1 and 2. Household income data were missing for 889 of 2148 participants (41.4%), leaving 1259 with observed income. The income-adjusted Model 3 included 1219 complete cases after all required covariates were considered.

### Primary associations

In the primary complete-case model, each 1-point increase in HSI was associated with 31% lower odds of sustained cessation (AOR=0.689; 95% CI: 0.631–0.752; p<0.001) (Table 2). Major smoking-related disease was independently associated with higher odds of sustained cessation after mutual adjustment (AOR=1.494; 95% CI: 1.087–2.054; p=0.013). The other chronic-condition count was not clearly associated with sustained cessation (AOR per condition, 1.083; 95% CI: 0.964–1.216; p=0.181). The estimates for HSI and major disease changed little when residence, education level, partnered status, and alcohol use were added to Model 1 to form the prespecified primary model (Model 2). Estimates were also similar in Model 3, which additionally included household income, and Model 4, which additionally included CESD-10 score and body mass index. In Model 4, the ΑORs were 0.666 (95% CI: 0.603–0.735) for HSI and 1.424 (95% CI: 1.010–2.008) for major disease. There was no evidence that the linear HSI cessation association differed by major disease status (interaction AOR=0.988; 95% CI: 0.817–1.194; p=0.897).

**Table 2:** Adjusted associations with sustained smoking cessation across model specifications in CHARLS, 2011–2018 (model-specific N=1219–2148)

*Factor*	*Model 1: Basic* *(N=2075)*	*Model 2: Primary* *(N=2075)*	*Model 3: Income-adjusted* *(N=1219)*	*Model 4: Health-adjusted* *(N=1760)*	*Model 2: MI* *(N=2148)*
HSI, per 1-point increase	0.689 (0.630–0.752)	0.689 (0.631–0.752)	0.689 (0.618–0.768)	0.666 (0.603–0.735)	0.687 (0.630–0.749)
Major smoking-related disease (yes vs no)	1.496 (1.089–2.053)	1.494 (1.087–2.054)	1.542 (1.032–2.302)	1.424 (1.010–2.008)	1.480 (1.082–2.025)
Other chronic conditions, per additional condition	1.085 (0.966–1.219)	1.083 (0.964–1.216)	1.110 (0.962–1.281)	1.100 (0.967–1.250)	1.103 (0.985–1.235)

Values are AORs (95% CIs). Model 1 included the three displayed variables, age, and sex. Model 2 additionally included rural residence, education, partnered status, and current alcohol use and was designated the primary model. Model 3 added inverse-hyperbolic-sine-transformed household income. Model 4 added CESD-10 score and body mass index to Model 2. Multiple imputation used the Model 2 covariate set. The HSI and major-disease estimates are mutually adjusted. CI, confidence interval; CESD-10, 10-item Center for Epidemiologic Studies Depression Scale; CHARLS, China Health and Retirement Longitudinal Study; HSI, Heaviness of Smoking Index; MI, multiple imputation; AOR, adjusted odds ratio.

### Functional form of HSI association

The restricted cubic spline showed evidence of both an overall association between HSI and sustained cessation (Wald χ^2^=121.08; df=3; p<0.001) and nonlinearity (Wald χ^2^=28.40; df=2; p<0.001). Relative to HSI=2, the adjusted odds ratio was 3.47 (95% CI: 2.51–4.80) at HSI=0 and 0.97 (95% CI: 0.56–1.70) at HSI=6 (Figure 2). The fitted curve declined sharply from HSI 0, reached its descriptive minimum near HSI 3.77, and then turned upward; estimates at the upper scores were imprecise and did not support defining a dependence cutoff.

**Figure 2:**
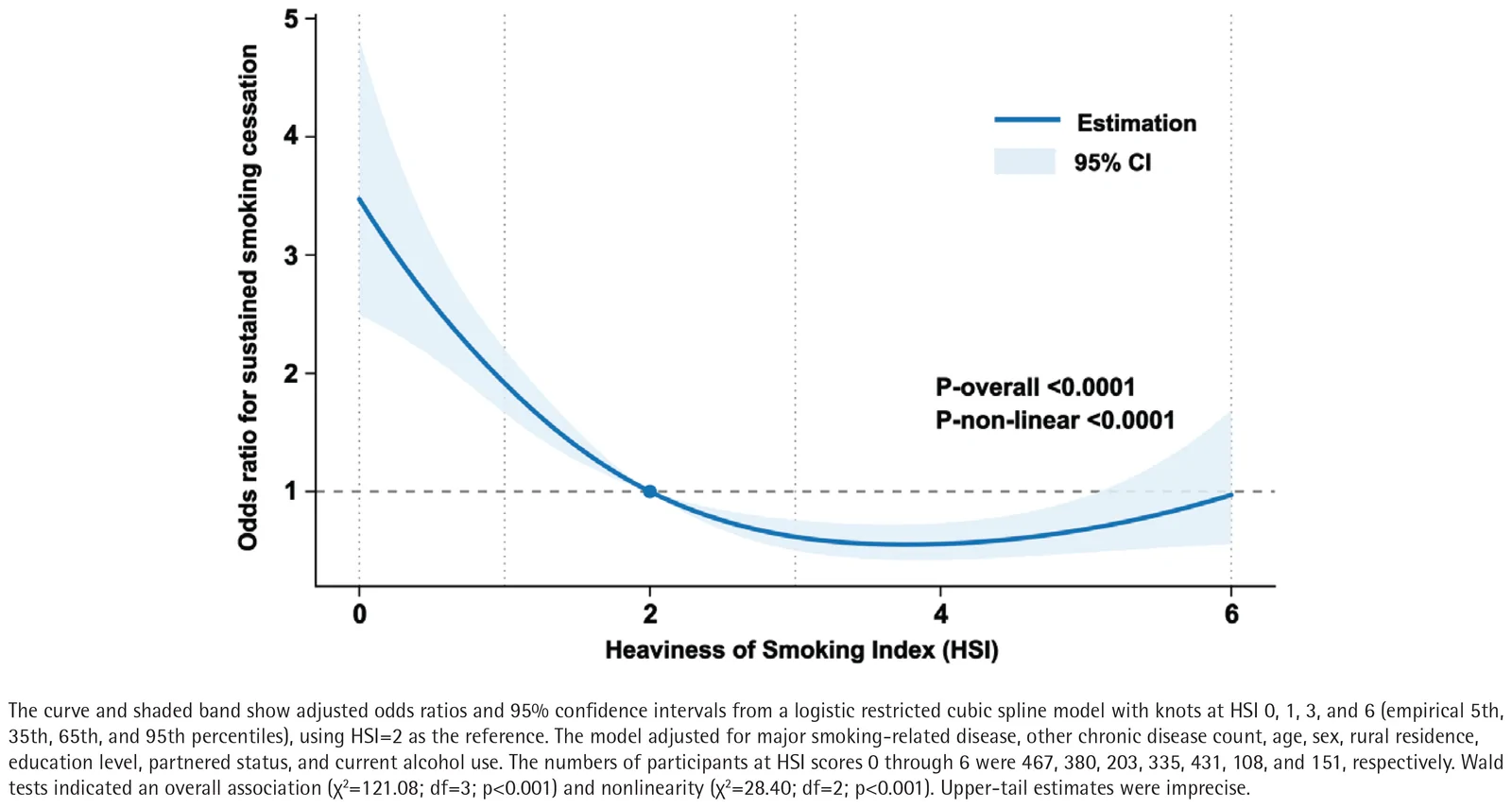
Adjusted odds-ratio curve for sustained smoking cessation by baseline HSI, CHARLS, 2011–2018 (primary complete-case sample, N=2075; 325 events)

### Sensitivity and smoking-transition analyses

Multiple imputation included all 2148 participants and 337 sustained-cessation events. Pooled estimates were similar to the complete-case results, with an AOR of 0.687 (95% CI: 0.630–0.749) per HSI point and an AOR of 1.480 (95% CI: 1.082–2.025) for major disease (Table 3). Adding imputed household income yielded corresponding ΑORs of 0.687 (95% CI, 0.631–0.749) and 1.492 (95% CI: 1.091–2.042). Attrition weighting also produced similar estimates, with an AOR of 0.682 (95% CI: 0.624–0.746) for HSI and 1.506 (95% CI: 1.094–2.072) for major disease. After truncation, the weights ranged from 0.792 to 1.589, and the effective sample size was 2020.

**Table 3:** Sensitivity analyses and secondary smoking-transition models in CHARLS, 2011–2018 (analysis-specific N=711–2148)

*Analysis*	*N/events*	*HSI per 1-point* *increase* *AOR (95% CI)*	*Major smoking-related* *disease* *AOR (95% CI)*	*Other chronic* *conditions* *AOR (95% CI)*
Primary complete-case model	2075/325	0.689 (0.631–0.752)	1.494 (1.087–2.054)	1.083 (0.964–1.216)
Primary multiple imputation	2148/337	0.687 (0.630–0.749)	1.480 (1.082–2.025)	1.103 (0.985–1.235)
MI with household income	2148/337	0.687 (0.631–0.749)	1.492 (1.091–2.042)	1.102 (0.983–1.234)
Attrition IPW	2075/325	0.682 (0.624–0.746)	1.506 (1.094–2.072)	1.077 (0.958–1.211)
Strict three-wave outcome, MI	711/63	0.599 (0.486–0.739)	2.284 (1.052–4.957)	1.201 (0.953–1.513)
Relapse vs persistent smoking	1902/152	0.744 (0.666–0.831)	1.293 (0.850–1.967)	1.030 (0.887–1.195)
Late cessation vs persistent smoking	2017/267	0.911 (0.845–0.983)	1.082 (0.754–1.550)	1.114 (0.994–1.248)

All models included HSI, major smoking-related disease, other chronic-condition count, age, sex, rural residence, education level, partnered status, and current alcohol use. IPW denotes stabilized inverse-probability weighting for an observed primary outcome, truncated at the 1st and 99th percentiles. The strict outcome compared non-smoking at all 2013, 2015, and 2018 waves with smoking at all three waves. Relapse and late cessation were each compared with persistent smoking in separate models. CHARLS: China Health and Retirement Longitudinal Study. HSI: Heaviness of Smoking Index. IPW: inverse-probability weighting. MI: multiple imputation. AOR: adjusted odds ratio. CI: confidence interval.

The stricter three-wave analysis included 711 participants, of whom 63 reported non-smoking in 2013, 2015, and 2018. In the imputed model, higher HSI remained associated with lower odds of sustained cessation (AOR=0.599; 95% CI: 0.486–0.739), whereas major disease remained associated with higher odds (AOR=2.284; 95% CI: 1.052–4.957). The estimate for major disease was less precise because this analysis included fewer sustained-cessation events. Relative to persistent smoking, each 1-point higher HSI was associated with lower odds of the sustained-cessation pattern (AOR=0.689; 95% CI: 0.631–0.752), relapse pattern (AOR=0.744; 95% CI: 0.666–0.831), and late-cessation pattern (ΑOR=0.911; 95% CI: 0.845–0.983). Major disease was associated with higher odds of sustained cessation, whereas its associations with relapse (ΑOR=1.293; 95% CI: 0.850–1.967) and late cessation (ΑOR=1.082; 95% CI: 0.754–1.550) were imprecise and included the null. Because these estimates came from separate binary models, differences in their magnitude were not formally tested across smoking-transition patterns. Figure 3 summarizes the primary, sensitivity, and smoking-transition analyses.

**Figure 3:**
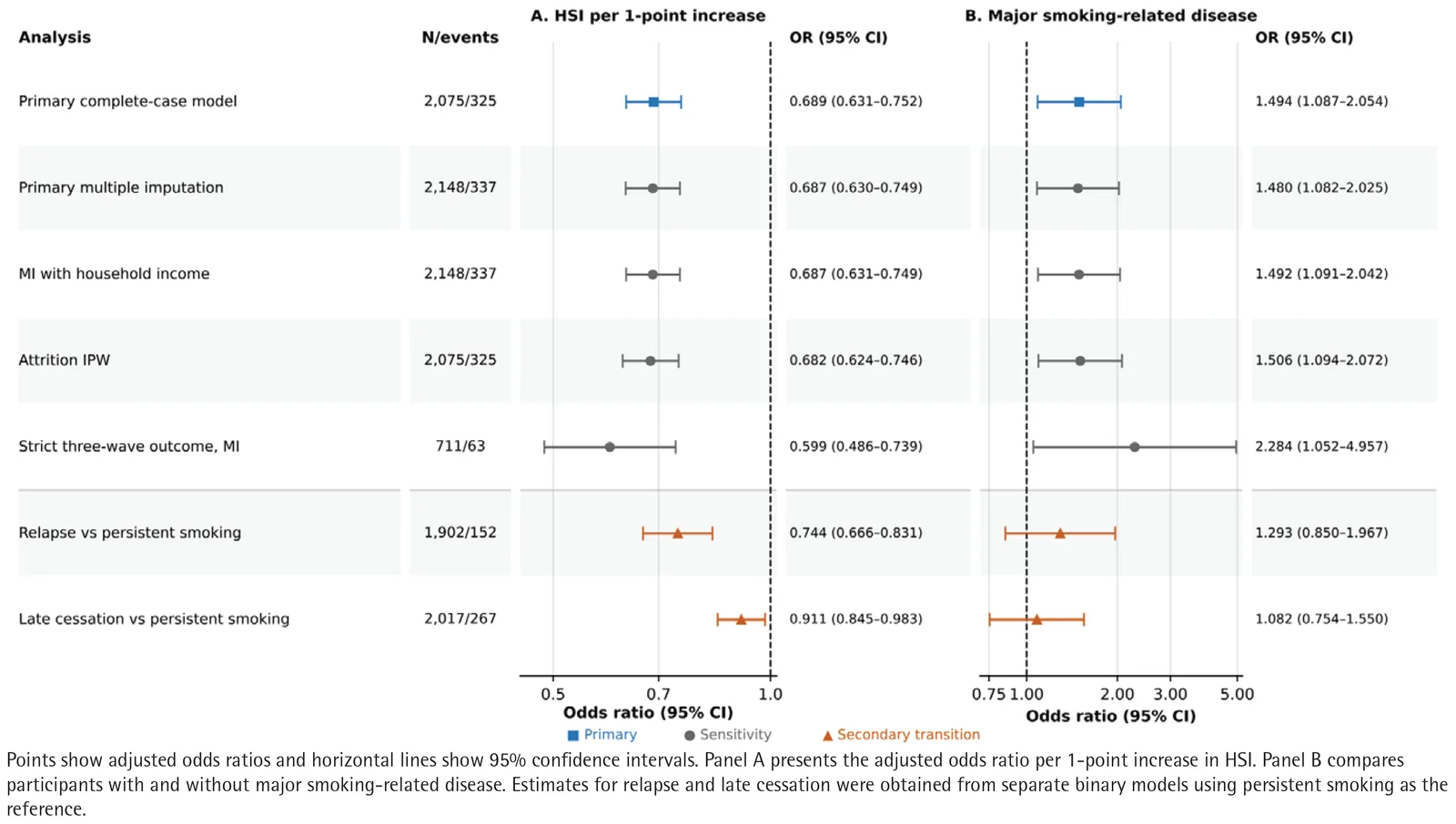
Adjusted associations of nicotine dependence and major smoking-related disease with smoking outcomes across the primary analysis, sensitivity analyses, and secondary smoking-transition models in CHARLS, 2011–2018 (analysis-specific N=711–2148)

### Exploratory effect modification

Exploratory interaction analyses are summarized in Supplementary file Table S1. There was no clear evidence that the HSI association differed by sex (p=0.203) or birth cohort (p=0.097), and the major-disease association did not clearly differ by birth cohort (p=0.597). Although the major-disease × sex interaction test yielded p=0.016, the estimate in women was based on only five sustained-cessation events among 50 women with major disease and was considered unstable.

## DISCUSSION

In this four-wave cohort of middle-aged and older Chinese adults who smoked at baseline, nicotine dependence and major smoking-related disease showed opposing associations with sustained cessation. Higher baseline HSI was associated with lower odds of reporting non-smoking at both later interviews, whereas heart disease, stroke, chronic lung disease, or cancer at baseline was associated with higher odds. These associations remained when the two factors were modeled together, and there was no evidence that the linear HSI cessation association differed by major disease status. Estimates were similar after multiple imputation, inverse-probability weighting for follow-up selection, use of a stricter three-wave cessation definition, and additional socioeconomic and health adjustment.

The HSI findings integrate observations that previous CHARLS studies examined separately. Qiu et al.^5^ found that fewer cigarettes smoked per day and a longer interval before the first morning cigarette predicted quitting, whereas earlier smoking after waking predicted relapse. Combining these items is important because cigarette consumption alone may incompletely capture dependence when smokers reduce consumption but continue to smoke soon after waking. More recent CHARLS research classified dependence using cigarette consumption alone and examined chronic disease as the outcome rather than sustained cessation^12^. The present analysis used the validated two-item HSI algorithm and required concordant non-smoking reports in 2015 and 2018. The adjusted spline curve declined sharply from HSI 0 through the mid-range and then rose at HSI 5–6, with wide confidence intervals in the upper tail. This imprecision argues against deriving a single dependence threshold from these data.

The association between major smoking-related disease and sustained cessation is compatible with, but does not establish, disease-related motivation to quit. In a quasi-experimental CHARLS analysis, incident diagnoses of cancer, heart disease, or stroke were followed by reductions in smoking, whereas associations with less severe diagnoses were smaller and less persistent^9^. Similar behavioral changes following illness have been reported in other cohorts^19,20^. Disease-related encounters have therefore been described as potential ‘teachable moments’ for behavior change^21^. However, CHARLS recorded prevalent diagnoses at baseline without information on diagnosis dates. Our analysis could therefore neither test this mechanism nor estimate the immediate causal effect of a health shock.

The major-disease association was specific to an indicator comprising heart disease, stroke, chronic lung disease, and cancer, whereas the count of ten other chronic conditions was not clearly associated with cessation. Disease severity or salience may be relevant, but this contrast should not be interpreted as evidence that these four diseases are inherently more motivating than other conditions. Alternative explanations include greater exposure to cessation advice, closer family supervision, symptoms that make smoking difficult, and unmeasured differences in healthcare use.

Considering dependence and disease together refines the practical interpretation. There was no evidence that the linear dependence gradient was weaker among participants with major disease. Major disease may therefore mark an opportunity for cessation support, but its presence should not be assumed to overcome dependence-related difficulty in maintaining abstinence. This distinction may help explain why persistent smoking remains common among older Chinese adults with smoking-related morbidity^10^, despite evidence that cessation is followed by better health indicators among people with chronic disease^22^.

From a population health perspective, brief dependence assessment could complement routine smoking-status assessment in chronic disease care. The HSI requires only two questions and could be recorded in cardiovascular, respiratory, oncology, stroke, and primary-care settings^6^. It may help identify smokers who warrant more detailed assessment, closer behavioral follow-up, and guideline-concordant cessation support^23^. However, this study did not evaluate treatment selection, predictive performance, or HSI-based intervention thresholds. Prospective studies could examine whether incorporating HSI assessment into chronic-disease care improves sustained abstinence and may help determine whether HSI-informed support adds value beyond routine smoking-status assessment. Exploratory subgroup findings should not guide practice. Women constituted only 8.3% of the analytic sample, and interaction testing was not adjusted for multiplicity. The estimates should therefore be regarded as hypothesis-generating.

### Strengths and limitations

This study has several strengths. Sustained cessation was distinguished from persistent smoking, relapse, and late cessation using repeated interviews. Dependence was measured using a validated composite rather than cigarette consumption alone. Major smoking-related disease and broader multimorbidity were modeled simultaneously using a prespecified conceptual framework. Multiple imputation, follow-up-selection weighting, flexible modeling of the HSI association, a stricter outcome definition, and transition-specific models examined different sources of analytic uncertainty. Complete-case and imputed estimates were closely aligned.

Several limitations define the scope of inference. First, smoking status, cigarette consumption, time to the first cigarette, and physician diagnoses were self-reported, creating potential information and misclassification bias. If misclassification was largely non-differential with respect to the modeled associations, it would generally be expected to attenuate estimates toward the null; differential reporting by disease or cessation status could bias estimates in either direction. Biochemical verification of abstinence and medical-record confirmation of disease were unavailable, and reporting non-smoking in both 2015 and 2018 also does not establish uninterrupted abstinence between interviews. Second, disease timing, smoking duration, previous quit attempts, cessation treatment, and reasons for quitting were not observed. We therefore cannot determine whether disease prompted cessation or identify the underlying mechanism. Third, the primary contrast was restricted to participants with concordant smoking states in 2015 and 2018. This created a clear comparison between sustained cessation and persistent smoking but excluded participants with relapse or late cessation from the primary analysis. Consequently, the analysis did not estimate the probability of any cessation among all baseline smokers. Fourth, the four-condition disease indicator combined illnesses with different symptoms, prognoses, and opportunities for cessation counseling. Because some component diseases, particularly cancer and stroke, were uncommon, separate disease-specific estimates would have been imprecise. Fifth, residual confounding by healthcare access, detailed socioeconomic circumstances, tobacco history, and family support remains possible. Household income was missing for 889 of 2148 participants (41.4%), although its inclusion after multiple imputation did not materially change the estimates. Attrition weighting can address only selection explained by observed baseline variables. Finally, the upper HSI levels and female smoking subgroups contained fewer sustained-cessation events, limiting interpretation of nonlinear and sex-specific estimates and generalizability of the findings to women.

Overall, major smoking-related disease may mark an opportunity to strengthen cessation care, but nicotine dependence remains relevant to the maintenance of abstinence. Prospective studies should evaluate whether incorporating brief HSI assessment into chronic disease care can improve sustained cessation and may help determine whether HSI-informed support adds value beyond routine smoking-status assessment.

## CONCLUSIONS

Within the primary sample of Chinese adults aged ≥45 years who had concordant smoking status in 2015 and 2018, greater nicotine dependence was associated with lower odds of sustained cessation versus persistent smoking, whereas major smoking-related disease was associated with higher odds after mutual adjustment. Major disease may identify an opportunity to offer cessation support, but there was no evidence that it weakened the inverse linear association between HSI and sustained cessation. Prospective studies could examine whether incorporating brief HSI assessment into chronic-disease care improves sustained abstinence and may help determine whether HSI-informed support adds value beyond routine smoking-status assessment.

## Supplementary Material



## Data Availability

CHARLS data are available to registered researchers through the project website (https://charls.pku.edu.cn/). The variable-construction and statistical-analysis R scripts used for this study are available from the corresponding author.
