# Supplementary material for: Nicotine dependence, major smoking-related disease, and sustained smoking cessation among middle-aged and older adults in China: A secondary analysis of the China Health and Retirement Longitudinal Study, 2011–2018
Source: Tob Induc Dis. 2026 Oct 22;24:10.18332/tid/240543. doi: 10.18332/tid/240543 (PMC13690070; doi:10.18332/tid/240543)

Tobacco Induced Diseases

Supplementary file

© 2026 Shi L. et al.

DOI:
10.18332/tid/240543

The content has been provided by the author(s) and has not been reviewed, verified, or endorsed by European Publishing. It may not have undergone peer review. The views, opinions, and recommendations expressed are solely those of the author(s) and do not necessarily reflect the position of European Publishing. European Publishing accepts no responsibility or liability for any consequences arising from the use of, or reliance on, this content.

Supplementary Table S1. Exploratory effect-modification analyses, China Health and Retirement Longitudinal Study (CHARLS), 2011-2018 (primary complete-case sample, N=2075)

Interaction	Global P value	Stratum-specific estimate or interpretation
HSI × sex	0.203	No clear evidence of effect modification.
Major disease × sex	0.016	Men: AOR=1.699 (95% CI: 1.238-2.332); women: AOR=0.334 (95% CI: 0.079-1.420). The female estimate was based on 5 events among 50 women with major disease and was unstable.
HSI × birth cohort	0.097	No clear evidence of effect modification.
Major disease × birth cohort	0.597	No clear evidence of effect modification.

Birth cohorts were 1945 or earlier, 1946-1955, and 1956-1966. No adjustment for multiple testing was applied. CI, confidence interval; HSI, Heaviness of Smoking Index; AOR, adjusted odds ratio.

Supplementary Figure S1. Conceptual framework for the mutually adjusted associations of nicotine dependence and major smoking-related disease with sustained smoking cessation in a secondary analysis of the CHARLS, 2011-2018

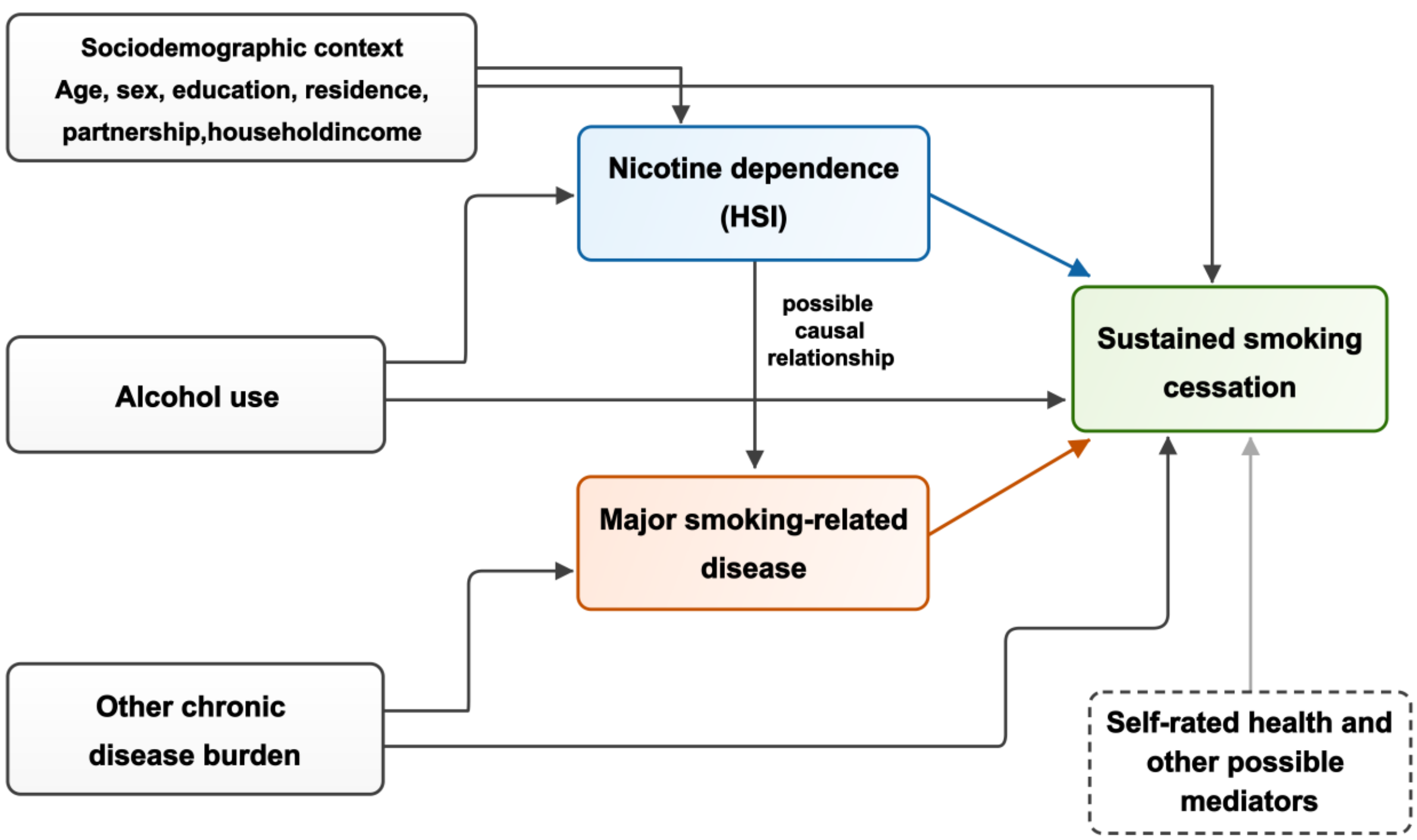

Supplement: Supplementary file 1 [file TID-24-193-s1.pdf]
